# Molecular Mechanisms of Bacterial Communication and Their Biocontrol

**DOI:** 10.3390/ijms25105443

**Published:** 2024-05-16

**Authors:** Corinne Barbey, Xavier Latour

**Affiliations:** 1 Laboratory of Bacterial Communication and Anti-Infectious Strategies (CBSA UR4312, Formerly LMSM EA4312), University Rouen Normandie, Université Caen Normandie, Normandie University, F-76000 Rouen, France; corinne.barbey@univ-rouen.fr; 2 Research Federation NORVEGE Fed4277, Normandie University, F-76000 Rouen, France; 3 International Research Federation NOR-SEVE, University of Sherbrooke, Sherbrooke, QC J1K 2R1, Canada; 4 Biocontrol and Biostimulation for Agroecology Association (ABBA), F-75000 Paris, France

A bacterium’s ability to colonize and adapt to an ecological niche is highly dependent on its capacity to perceive and analyze its environment and its ability to interact with its hosts and congeners. It has long been believed that each bacterial cell represents an autonomous individual that is free to move, whereas bacteria are social organisms that develop a multitude of cooperative and competitive interactions, which are essential for their becoming. These interactions are commonly observed at the species scale, but also within the microbial community, such as that encountered in a biofilm. They are supported by bacterial communication, which is the exchange of information between cells. The aim of this Special Issue was to contribute to the existing research addressing some molecular mechanisms of environmental sensing (perception of abiotic and biotic factors), bacterial communication, and/or their control, leading to mechanistic or applied progress.

Communications are mediated through the exchange of signals. According to Venturi and Keel [1], a signal is a mobile compound whose occurrence leads to one or more cellular responses by the receiving cell that are not limited to catabolism, transformation, or other aspects of this compound (e.g., resistance to its toxicity). Nevertheless, a semantic distinction can modulate this definition to better characterize the determinism of interactions between bacteria and their environment or between bacteria themselves. To clarify the role of the suspected signaling compound, it is necessary to show whether or not the interaction between speakers evolved as a response to this compound. The word “cue” is usually attributed to a compound (only) responsible for the perception of the environment, while the term “signal” refers to a real exchange of information between partners, providing a fitness benefit to both the sender and receiver [2]. If the cues can be of great diversity (photons, ions, molecules, temperature change, etc.), the signals are generally small diffusible molecules that are often amphiphilic. As a result, the latter easily cross the cell envelope of the emitting cell and then freely diffuse through the aqueous or aerial environment depending on whether the signaling molecules are volatile. Sometimes, signals also have an action on their producer and are auto-inducers (AIs) of their own synthesis [3]. The signals most described in the Gram-negative bacteria bibliography belong to the *N*-acyl-homoserine lactone (AHLs) family, so-called type 1 autoinducers (AI-1). AHLs are composed of a homoserine lactone ring, a marker of the signaling nature of the molecule, and an acyl chain, whose variability ensures the specificity of communications [3]. AHLs’ exchange-based communication is the subject of a study published in this Special Issue [4]. Other diffusible signals have been identified in Gram-negative bacteria, such as the diffusible signaling factors (DSFs) in *Xanthomonas* spp.; 4-hydroxy-2-heptylquinoline (HHQ) and 3,4-dihydroxy-2-heptylquinoline (PQS), which are produced by members of the *Pseudomonas aeruginosa* species and certain *Burkholderia* spp.; or fatty acid derivatives (3-OH-PAME and 3-OH-MAME) produced by *Ralstonia* spp. [5,6]. In Gram-positive bacteria, the signals are generally small peptides, except in Actinobacteria, which exchange signals belonging to the γ-butyrolactone family [7,8]. Finally, type 2 autoinducers (AI-2) and diketopiperazine (DKP) production is shared by bacteria belonging to one of the Gram stains, such as members of the *Pseudomonas*, *Streptomyces*, or *Bacillus* genera [3,9]. 

At the other end of the communication network, the reception of information takes place on membrane or cytosolic receptors according to the lipophilicity of the signaling compounds. Membrane sensors, such as two-component systems (TCSs), are powerful tools that not only fulfill the environmental conditions in which the cell is immersed but are also responsible for perceiving extracellular cues emitted by the eukaryotic host or prokaryotic congeners. TCSs are widespread among bacteria because of their versatility and the variety of the recognized cue/signal. Various TCS families’ molecular functioning structures and subtleties have previously been reviewed in detail [10,11]. In summary, a TCS consists of a sensor kinase embedded in the inner membrane and a cytosolic cognate response regulator. After encountering its environmental biotic or abiotic ligand, the sensor undergoes autophosphorylation and activates the dedicated cytosolic response regulator by phosphotransfer. Several articles in this Special Issue have contributed to characterizing the diversity of TCSs, particularly that of histidine kinase sensors (HKs) [12,13]. Here, HKs play an essential role in the detection of nitrous compounds and certain organic acids of the Krebs cycle. These molecules, which are electron acceptors and donors, respectively, act as cues by modulating the functioning of the bacterial primary metabolism and by providing information on the presence of competing neighboring cells.

Bacteria use cell-to-cell communication systems based on the synthesis and perception of signaling molecules to evaluate their density and synchronize their social behavior. For example, quorum-sensing (QS) systems control diverse functions, which require the concerted actions of numerous cells in order to be productive, such as antibiotic synthesis, motility, symbiosis, sporulation, virulence, and biofilm formation [3,14]. Indeed, bacteria are rarely encountered as single dispersed organisms in the environment. They generally live in communities and may colonize the surfaces of minerals and living tissues by forming biofilms [14,15,16]. A biofilm is a dynamic and organized microbial community that consists of heterogeneous cells/populations evolving in time and space [17]. The strong genetic heterogeneity within a biofilm promotes dynamic evolution in the appearance of positive and negative social interactions. The results of these interactions include the fitness modification of the surrounding cells, the production of public goods, and the modification of the biofilm structure [18]. These modifications are explained by the rise in cooperative behavior between certain cells within the biofilm. Thus, the biofilm phenotype may be seen as a form of collective behavior in which all of the members of the community work together to ensure the persistence of the group within the environment. For example, bacteria express different phenotypes according to their location within the structure of the biofilm; the bacteria at the periphery are in an active metabolic state, enabling them to shield the “core bacteria” at the center of the biofilm, including dormant persister cells [19]. Cooperative interactions require finely tuned coordination between partners. This coordination is mediated by bacterial communications which are obviously encountered within biofilm. In this Special Issue, two works reveal the roles of AHL- and peptide-signaling molecules in the switching of bacteria between motile behavior and a sessile lifestyle, leading to biofilm formation and maturation [4,20]. Thus, it is known that the *Pseudomonas putida*’s lifestyle is complexly regulated by several cellular factors. Among them, its cell surface adhesin LapA is a major determinant for attachment and biofilm formation. Extracellular peptides contained in tryptone growth media enhance *P. putida* biofilm formation not because they serve as nutrients, but because they impact *lapA* expression [20]. AHL-based QS networks are also strongly suspected to function as master regulators of biofilm development in many Gram-negative bacteria. Indeed, each individual who communicates constitutively produces diffusible AHL signals, the environmental concentrations of which are directly modulated by the level of cell lockdown. Biofilm members that can detect AHLs are informed about the number of neighbors and the degree of diffusion from their microenvironment, namely whether they are located at the core, at the periphery, or outside the biofilm. QS also enables bacteria to synchronize the expression of genes involved in biofilm synthesis, the production of public goods, and other cooperative traits with benefits available to all the cells of the population for a minimal individual cost [4]. Here, AHLs should be considered both cues and signals. Indeed, during the exponential growth phase of transmitter cells, AHLs act as cues informing the population about the cell density, while at the beginning of the stationary phase, they become signals by generating a change in the behaviors of emitter and receiver bacteria [2]. 

The existence of “communication quenchers” among the other organisms that live alongside communicating populations highlights a major weakness in the establishment or persistence of social behavior when these are strongly controlled by communication systems such as QS systems. In the early 2000s, Dong and colleagues discovered a *Bacillus* sp. strain capable of degrading AHLs. This inactivation was attributed to the lactonase AiiA (autoinducer inhibition A), which can open the lactone bridge of AHLs, modifying their structure and their signal function [21]. The authors then presented the original idea that using this enzyme by constructing transgenic plants, for example, would make it possible to disrupt the communication of phytopathogens using AHLs as signals. A few years later, it was shown that the bacteria producing AHL signals represented 10 to 20% of the cultivable bacteria in the soil and the rhizosphere and that the bacteria capable of degrading these AHLs represented 5 to 10% of the cultivable bacterial populations in these same niches [22]. This suggests that information jamming, which is carried out by certain hosts or antagonistic populations, is common in the environment and that it can participate in the communication process by modulating or masking it from interested populations. Moreover, the functioning of QS communication can be disrupted by higher organisms that have co-evolved alongside signal-emitting bacterial populations. Indeed, certain plant or animal hosts can cleave signals, potentially disrupting the QS signaling of associated bacteria [23,24]. Finally, certain animals and plants can manufacture molecules similar to signaling molecules, which can mislead or inhibit QS communication through this mimicry. This is the case of the red alga *Delisea pulchra*, which produces halogenated furanones, but also of higher plants producing derivatives of indole signals and flavonoids [24,25,26]. This interregnum communication is also perceptible in articles published in this Special Issue relating the interactions between human pathogens, such as the strong producers of biofilms *Pseudomonas aeruginosa* and *Escherichia coli*, with their infected hosts [27,28]. Some determinants of these pathogens are detected as cues that trigger a coordinated host response. These cues can modulate the humoral innate immunity of the host by interfering with Imd and Toll-signaling cascades. 

Therefore, communication quenching strategies can help protect the host when bacterial behavior switches as a result of host detection with the aim of infecting it, colonizing it and triggering the disease. This can be carried out using biocontrol therapies based on the use of beneficial microorganisms or their by-products targeting signaling production or reception used by the pathogenic bacteria [24,29,30,31]. These innovative anti-virulent strategies are currently being developed.
